# A Possible Role for Idiotype/Anti-idiotype B–T Cell Interactions in Maintaining Immune Memory

**DOI:** 10.3389/fimmu.2017.00409

**Published:** 2017-04-06

**Authors:** Victor I. Seledtsov, Galina V. Seledtsova

**Affiliations:** ^1^Immanuel Kant Baltic Federal University, Kaliningrad, Russia; ^2^Institute for Fundamental and Clinical Immunology, Novosibirsk, Russia

**Keywords:** immune memory, memory B-cell, memory T cell, B-cell receptor, T-cell receptor, idiotype/anti-idiotype interaction

## Abstract

Variable regions of both B-cell receptors (BCRs) and T-cell receptors (TCRs) are completely formed in the postnatal period, and, consequently, no innate immune tolerance against these structures exists in adulthood. Indeed, antibodies (Abs) specific to TCRs have been found in both animals and humans. These facts clearly indicate the existence of B cells able to directly interact with T cells through binding of BCRs to TCRs without implicating major histocompatibility complex molecules. A novel paradigm is proposed in that the immune memory is based on idiotype/anti-idiotype interactions occurring between BCRs and TCRs following clearance of the antigen that elicited immune responses. It is envisaged that direct contact between memory T and B cells could provide co-stimulatory signals needed to sustain viability, growth, and differentiation of the interacting immune cells. In contrast, plasma cells originating from memory B-cells could produce anti-TCR Abs that inhibit direct BCR-to-TCR interactions, thereby downregulating the B- to T-cell contact-based immune memory *via* a negative feedback mechanism.

## Introduction

Acquired immunity is well known to facilitate the recognition of target antigens (Ags) and the development of more rapid and effective immune responses upon secondary Ag exposure. Mature T and B lymphocytes are central cellular mediators of specific immunity. Upon infection or vaccination, T-cell receptors (TCRs) recognize antigenic peptides bound to major histocompatibility complex (MHC) molecules on the surface of antigen-presenting cells. TCR signals cooperate with cytokine, co-stimulatory, chemokine, integrin, and metabolic signals to stimulate generation of memory T cells of different phenotypes (1, 2). Unlike TCRs, B-cell receptors (BCRs) recognize intact Ags not requiring Ag processing and implication of MHC molecules for Ag presentation. However, B-cell differentiation and generation of memory B cells require continuous co-stimulatory molecule-dependent and cytokine-dependent help from Ag-reactive T cells (3). Several lines of evidence have shown that memory T and B cells are major components of long-lasting anti-infectious immune protection status (2, 4, 5). The persistence of immune memory is arguably best exemplified by the finding that Ag-specific T cell responses can still be identified 60 years following vaccination against smallpox when antigenic re-encounter was completely excluded (2, 6). Immune responses to yellow fever vaccine provide further evidence that immune memory is long-lasting; indeed, immunity to this vaccine was shown to last for up to 35 years (2, 7, 8).

However, it still remains unclear how and why memory T and B cells survive for a long period of time in the absence of the cognate antigenic stimulus. The published data suggest a role for homeostatic cytokines, such as IL-7 and IL-15, in memory T and B cell survival (9). However, the absence of IL-7 or/and IL-15 only reduces, but not abrogates completely, the generation of the immune memory in different experimental models (9–11). From this, it appears that some memory cells can be generated and maintained in the absence of homeostatic cytokines.

Since variable BCR and TCR regions are extremely diversified, we propose that TCRs could elaborate three-dimensional antigenic images for BCRs and *vice versa* and that direct interactions between TCRs and BCRs could underlie long-term maintenance of the immune memory.

## Paradigm for B–T Cell Interaction in Maintaining Immune Memory

Mature lymphocytes express unique antigenic receptors, the functional form of which results from the random rearrangement of mini-gene segments, imprecise joining of nucleotide sequences, and random combinations of peptide chains. Although the human genome contains <25,000 genes, this developmental process can produce well over 100 million different Ag-binding specificities (12). Variable regions of BCRs and TCRs carry unique antigenic determinants that are called idiotypes (Ids). Since variable regions of Ag receptors are completely formed in the postnatal period, there is no innate immune tolerance against these molecules in adulthood. Indeed, Id determinants have been shown to be immunogenic and capable of eliciting anti-idiotype (anti-Id) immune responses (13–15). Variable regions of Ag receptors of Id-reactive lymphocytes are called anti-Id. Jerne’s network theory postulates that immune system functions as a regulatory network, which is based on Id/anti-Id interactions occurring between lymphocytes. The original network theory dealt only with immunoglobulins (Igs) with little reference to T cells (13). However, thymectomized (16) and nude (17) mice failed to produce anti-Id antibodies (Abs) in response to immunization with Id + Igs suggesting that Ig molecules are in fact T-cell dependent Ags. Indeed, in addition to inducing Abs, Ids/anti-Ids interactions also induce T cell responses. Such studies suggest that T cells need to be integrated into Id/anti-Id regulation network as well (18). In this context, it is tempting to rationalize that B cells present Id-derived peptides to Id-specific T cells in an MHC-restricted manner. Accordingly, Id-specific T cell clones have been shown to be capable of recognizing Id determinants in complexes with MHC class II molecules on the surface of B cells. Importantly, activation of B cells *per se* enables Ag presentation of both exogenous Ags and BCR-derived Id determinants to T cells (19, 20). In this case, B-cell induced TCR-mediated T-cell activation could promote generation of memory T cells, but not memory B-cells as membrane-associated BCRs remain uninvolved in Id/anti-Id immunoregulation.

**Figure 1:**
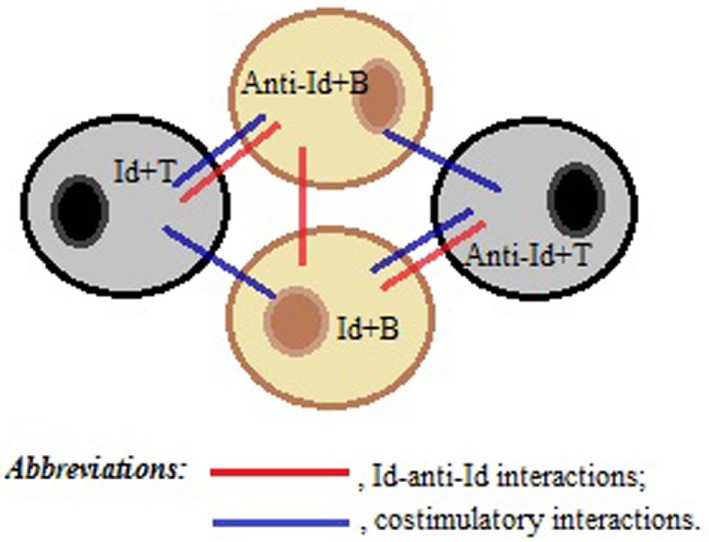
**A schematic representation of a T–B cell cluster responsible for the immune memory**. Direct idiotype (Id)–anti-idiotype (anti-Id) interactions between T- and B-cells, as well as between B- and B-cells, favor membrane and cytokine co-stimulations of both Id+- and anti-Id+ immune cells, thereby maintaining the viability of each other in the absence of antigenic stimulation.

We speculate that plasticity of BCR and TCR repertoires and structural similarities of Ag receptors in B and T cell compartments are important prerequisites that can facilitate contact and communications between B- and T-cells through direct Id/anti-Id BCR–TCR interactions. Furthermore, we hypothesize that some TCRs could form three-dimensional antigenic images recognizable by BCRs, while some BCRs with certain Id/anti-Id specificities could directly activate specific T cells. Thus, the nature of Id/anti-Id T- to B-cell collaboration could be bidirectional. We propose that upon TCR-induced BCR-mediated activation, B cells could upregulate the expression of co-stimulatory molecules, such as CD40, CD80, and CD86, thereby gaining strong T cell activation potential (21, 22). On the other hand, upon BCR-induced TCR-mediated activation, T-cells could upregulate the expression of CD40L and CD28 and provide cytokine-mediated, short distance co-stimulatory signals to B-cells. For example, such processes occur in T-cells under influence of cross-linking TCRs by particle-conjugated anti-CD3 Ab (23). As illustrated in Figure 1, contact-dependent bidirectional signaling could provide survival benefits for contacting lymphocytes in the absence of inflammation or lymphopenia when significant levels of any soluble viability factors including homeostatic cytokines are lacking in lymphocyte microenvironment. On the other hand, the functionality of a cluster consisting of interacting Id+- and anti-Id+-lymphocytes should be very plastic and extremely sensitive to the particular Ag that induced cluster formation in the first place.

**Figure 2:**
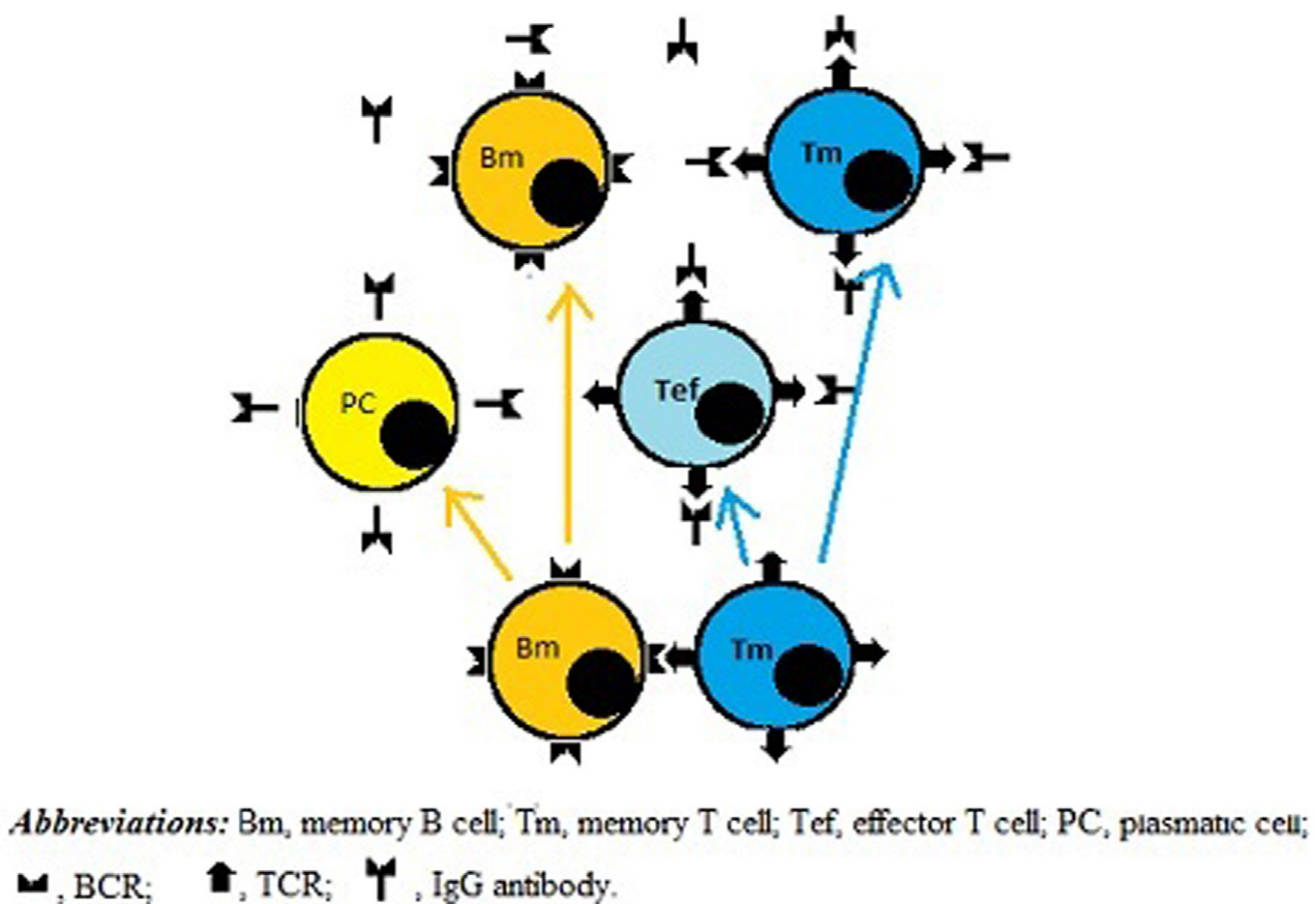
**A role for IgG antibodies (Abs) in downregulating immune memory**. Direct BCR-to-TCR interactions lead to growth and differentiation of memory B and T cells. Subsequently, plasma cells originated from memory B cells produce IgG Abs, which shield TCRs, thereby inhibiting not only growth and differentiation activity of memory B and T cells but also the functionality of effector T cells, including helper T cells.

According to our novel paradigm for B–T cell interaction, upon clearance of the exogenous Ag from the body the survival of Ag-specific Id-bearing memory T and B cells would depend on the presence of anti-Id B- and T-cells, respectively. In this model, direct BCR–TCR interactions leading to their cross-linking, together with co-stimulatory signals, could provide both growth and differentiation stimuli for individual B- and T-cells. As a result, new memory B- and T-cells, as well as new effector T-cells and plasma cells could be generated and further implicated in Id/anti-Id immunoregulation network. As illustrated in Figure 2, by shielding TCRs and preventing their cross-linking with BCRs, plasma cell-derived TCR-specific Abs could downregulate memory cell expansion. In addition, TCR-specific Abs present at high concentrations could also induce apoptosis in target T cells or kill them *via* complement- and/or FcR-dependent mechanisms. Consistent with this scenario, there are published data to suggest that anti-Id TCR-specific Ab responses can be induced by T cell vaccination in multiple sclerosis patients (24) and that these responses are likely to contribute to the suppression of myelin basic protein-reactive T cells in vaccinated patients (25). Moreover anti-TCR Abs have been proposed to prevent miscarriages and/or preterm births by downregulating maternal T-cells directed against HLA-DR molecules of the fetus (26).

The proposed paradigm for B–T cell interaction can be further developed to include other cellular mechanisms involved in controlling memory cell compartment, such as CD8+ effector cells capable of destroying B-cells through TCR-to-BCR interactions, which could be implicated in the downregulation of immune responses. Moreover, anti-Id Abs could have a significant effect on Id+ B-cell-mediated responses. Thus, the immune memory could be regulated by continuously working feedback mechanisms. We hypothesize that reduction in growth and differentiation activity of memory cells would lead to a decrease in plasma cells producing TCR-binding Abs, with the subsequent strengthening of BCR–TCR interactions and enhancement of growth and differentiation activity of immune cells responsible for the immune memory. Re-entry of Ag into the body should also upregulate the immune memory both by direct stimulation of memory B cells and reduction in Ag/TCR-specific Ab levels.

The novel paradigm proposed here envisages that primary immune responses lead to attaining the particular quantitative levels of Ag-specific lymphocytes necessary and sufficient for the induction of Id-specific immune responses and subsequent generation of stable Id/anti-Id T–B interactions responsible for immune memory formation. In this model, potentially auto-aggressive lymphocytes are initially depleted or suppressed by immunoregulatory mechanisms. Consequently, under normal conditions, a self-reactive lymphocyte clone is unlikely to achieve the quantitative threshold level necessary to generate functionally significant Id/anti-Id T–B cell interactions. However, should this level be reached, this situation could generate the pathological immune memory leading to the development of autoimmune diseases. According to our concept, an immunetheraupeutic approach based on vaccination with self-reactive T-cells could focus on the stimulation of Id-specific Abs shielding self-reactive TCRs. In this context, we also propose an important role of “ natural” T-cell vaccination in preventing autoimmune diseases. Indeed, pronounced auto-aggressive lymphocyte expansion caused by a particular Ag stimulus could induce immune memory *via* a B- and T-cell contact mechanism and subsequently stimulate the production of a large amount of anti-TCR Abs blocking effectively the activity of immune memory-based, auto-aggressive mechanisms. To our opinion, a largely similar mechanism could also underlie high-dose Ag-specific tolerance.

We also hypothesize that the development of IgE-mediated allergies could be, at least in part, due to FcR-mediated, high affinity adsorption of IgE by cells, resulting in their negligible ability of inactivate the particular anti-Id TCRs involved in the upregulation of allergic responses. Should this be the case, conventional allergen-specific therapy could ameliorate allergic process through inducing production of allergen-specific IgG Abs capable of shielding effectively such TCRs.

## Conclusion

The paradigm formulated in this paper suggests that the long-term maintenance of the immune memory occurring in the absence of the Ag that induced adaptive immunogenesis is based on the immunogenicity of BCRs and TCRs, as well as on the ability of memory B and T cells to support closely growth and differentiation activity of each other in a contact-dependent manner.

## Author Contributions

VS is responsible for the main idea and writing of the manuscript. GS is responsible for making the figures and reference list.

## Conflict of Interest Statement

The authors declare that the research was conducted in the absence of any commercial or financial relationships that could be construed as a potential conflict of interest.

## Abbreviations

Ab, antibody; Ag, antigen; anti-Id, anti-idiotype; BCR, B-cell receptor; Ig, immunoglobulin; MHC, major histocompatibility complex; TCR, T-cell receptor.
